# Recent advances in plant-herbivore interactions

**DOI:** 10.12688/f1000research.10313.1

**Published:** 2017-02-08

**Authors:** Deron E. Burkepile, John D. Parker

**Affiliations:** 1 Department of Ecology, Evolution, and Marine Biology, University of California Santa Barbara, Santa Barbara, CA, USA; 2 Marine Science Institute, University of California Santa Barbara, Santa Barbara, CA, USA; 3 Smithsonian Environmental Research Center, Edgewater, MD, USA

**Keywords:** plant defense theory, herbivore diversity, climate impact, plant-herbivore interactions

## Abstract

Plant-herbivore interactions shape community dynamics across marine, freshwater, and terrestrial habitats. From amphipods to elephants and from algae to trees, plant-herbivore relationships are the crucial link generating animal biomass (and human societies) from mere sunlight. These interactions are, thus, pivotal to understanding the ecology and evolution of virtually any ecosystem. Here, we briefly highlight recent advances in four areas of plant-herbivore interactions: (1) plant defense theory, (2) herbivore diversity and ecosystem function, (3) predation risk aversion and herbivory, and (4) how a changing climate impacts plant-herbivore interactions. Recent advances in plant defense theory, for example, highlight how plant life history and defense traits affect and are affected by multiple drivers, including enemy pressure, resource availability, and the local plant neighborhood, resulting in trait-mediated feedback loops linking trophic interactions with ecosystem nutrient dynamics. Similarly, although the positive effect of consumer diversity on ecosystem function has long been recognized, recent advances using DNA barcoding to elucidate diet, and Global Positioning System/remote sensing to determine habitat selection and impact, have shown that herbivore communities are probably even more functionally diverse than currently realized. Moreover, although most diversity-function studies continue to emphasize plant diversity, herbivore diversity may have even stronger impacts on ecosystem multifunctionality. Recent studies also highlight the role of risk in plant-herbivore interactions, and risk-driven trophic cascades have emerged as landscape-scale patterns in a variety of ecosystems. Perhaps not surprisingly, many plant-herbivore interactions are currently being altered by climate change, which affects plant growth rates and resource allocation, expression of chemical defenses, plant phenology, and herbivore metabolism and behavior. Finally, we conclude by noting that although the field is advancing rapidly, the world is changing even more rapidly, challenging our ability to manage these pivotal links in the food chain.

## Introduction

**Figure 1.**
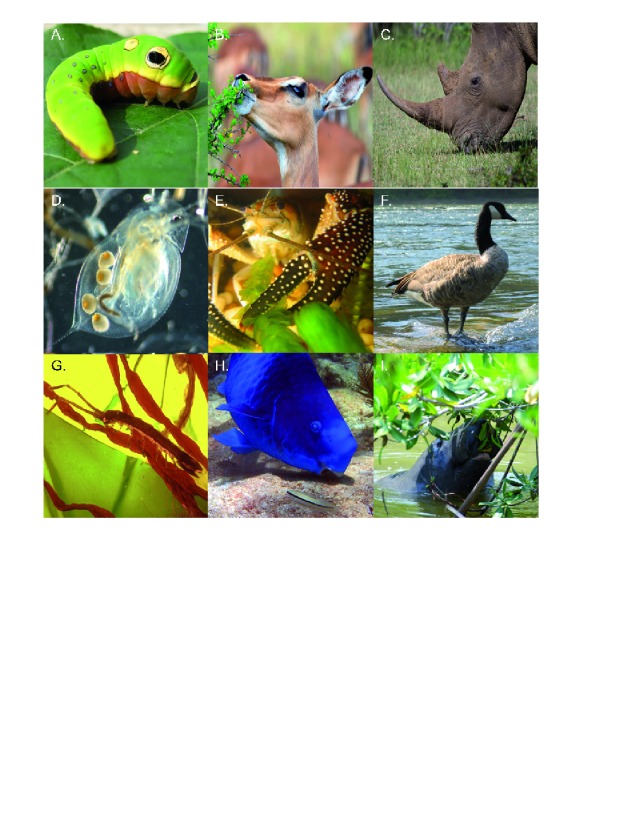
Plant-herbivore interactions shape community dynamics across terrestrial, freshwater, and marine habitats. These interactions encompass a huge range of taxa and organismal size for both plants and herbivores. Common herbivores across these ecosystems are ( **A**) spicebush swallowtail caterpillar ( *Papilio troilus*) feeding on spicebush ( *Lindera benzoin*); ( **B**) impala ( *Aepyceros melampus*) browsing shrubs in an African savanna; ( **C**) white rhinoceros ( *Ceratotherium simum*) grazing African grasses; ( **D**) *Daphnia dentifera*, an important grazer on freshwater phytoplankton; ( **E**) the white tuberculed crayfish ( *Procambarus spiculifer*) consuming a freshwater macrophyte ( *Egeria densa*); ( **F**) Canada goose ( *Branta canadensis*), an important herbivore on freshwater macrophytes and terrestrial grasses; ( **G**) the isopod *Erichsonella attenuata*, a mesograzer of epiphytes and seaweeds in seagrass meadows; ( **H**) blue parrotfish ( *Scarus coeruleus*) grazing filamentous algae on a coral reef; and ( **I**) West Indian manatee ( *Trichechus manatus*) curiously eating a red mangrove plant ( *Rhizophora mangle*). Photo credits: Eric Lind ( **A**), John Parker ( **B**, **E**, **F**, **G**, **I**), Deron Burkepile ( **C**), Alan Wilson ( **D**), Thomas Adam ( **H**).

Plant-herbivore interactions are important for understanding community dynamics and ecosystem function given that they are the critical link between primary production and food webs. Plant-herbivore studies are also the backbone of multiple fields within ecology and evolution, including co-evolution ^1, 2^, chemical ecology ^3– 5^, nutritional ecology ^6, 7^, and ecological stoichiometry ^8– 10^. The topic crosses ecosystem boundaries (freshwater, terrestrial, and marine) ^11^, huge ranges of organismal size (aphids to elephants and phytoplankton to trees) ^12, 13^ ( Figure 1), and vast productivity gradients (deserts to tropical rain forests) ^14– 16^, resulting in broadly applicable ecological theories.

Recent technological and statistical advances have resulted in a rapidly advancing field of study, including (1) the genetic and phylogenetic basis of plant-herbivore interactions and chemical defenses ^17– 21^, (2) DNA barcoding to elucidate herbivore diets ^22, 23^, (3) Global Positioning System (GPS) and remote sensing technology to understand landscape-level predator-herbivore-plant interactions ^24^, and (4) statistical advances that allow for comprehensive analyses of multiple co-varying drivers in both observational and experimental studies ^25^. Given the array of topics comprising the study of plant-herbivore interactions, our focus was not to exhaustively review this literature. Instead, we point out several exciting and growing areas of research from the past 3 years. We focus on briefly highlighting four areas of plant-herbivore interactions: (1) plant defense theory, (2) herbivore diversity and ecosystem function, (3) the context dependency of herbivory and predation risk, and (4) how a changing climate impacts plant-herbivore interactions. We strived to include a broad array of examples from across ecosystems and, in the process, are likely to have missed many worthy studies. We conclude that the study of plant-herbivore interactions continues to be a leading driver for ecology and evolution in general and that these studies can be used to inform conservation but that efforts need to be redoubled to counter the rapidly changing dynamics of the Anthropocene.

## Plant defense theory

The study of plant defense against herbivores is one of the cornerstones of ecology and evolution, underpinning the theory of co-evolution ^1^, the field of chemical ecology ^26^, and some of the most prominent mechanisms explaining the success of invasive species ^27– 29^. Recent work, however, has challenged some of the key paradigms in these fields, invigorating and broadening a variety of research perspectives. For example, invasive plants have long been thought to succeed via enemy loss (that is, the enemy release hypothesis), but direct evidence for this idea has been alternately supportive ^30, 31^, conflicting ^32, 33^, and ambivalent ^34^. Similarly, although chemical novelty from native species is one of the key mechanisms thought to drive enemy release ^35^, limited experimental tests show little relationship between novel chemistry and herbivore deterrence ^36^. Furthermore, enemy release is often unassociated with either increased invasiveness ^37– 39^ or competitive effects on neighboring plants ^40^. In light of these results, recent work has emphasized that we should be examining whether enemy release interacts with other ecological drivers, including resource availability or disturbance ^31, 41, 42^, and whether the complexity and integrity of the entire recipient food web are better predictors of invasion resistance than plant-herbivore interactions alone ^43^.

Similarly, plant chemical defenses have long been regarded as a primary source of defense against herbivores ^44^, and a host of articles have demonstrated the advantages of plant chemical defenses, particularly when examined in a comparative framework across model taxa (for example, ^45^). Nevertheless, when examined at the community scale across sympatric species, the relationships between chemical defense and herbivory are often weaker ^46, 47^. Some of this discrepancy might be due to the difficulty of comparing defense levels across disparate species with a diversity of chemical defenses that make direct comparisons problematic ^48^. However, the ambiguity between comparative and community-level studies highlights a recent re-emphasis of the importance of having mixed defensive strategies against a range of consumer types ^49, 50^. For example, variation in chemical defenses can interact with life history traits, structural defenses, nutrient quality, and the relative distribution of above-versus-belowground chemical defenses to modulate herbivory ^51– 56^, highlighting the need to examine the efficacy of chemical defenses within the context of a shifting mosaic of plant traits.

For example, theory predicts that low nutrient quality should lead to linear reductions in herbivory ^57, 58^, but Wetzel *et al.* ^7^ found that variance in nutrient traits (for example, protein and nitrogen content, among others), not low nutrient quality per se, determined the performance of 53 insect herbivore species. Insect performance increased when tissue nutrient levels were low but eventually declined when nutrients were high, likely due to either nutrient toxicity or even nutrient-toxin interactions (for example, ^59^), whereas increasing defenses nearly always led to linear declines in insect performance. Interestingly, relatively homogenous nutrient levels could provide a mechanistic explanation for why crops and some species-poor natural communities might be more prone to insect outbreaks when compared with diverse systems ^60, 61^. Moreover, these findings generally mirror those from research focusing on plant traits, as variance in plant traits appears to be just as important as mean trait levels if not more so ^62^.

Numerous studies have also demonstrated that plant-herbivore interactions are context-dependent, modulated by the surrounding plant community ^63– 68^, by local nutrient conditions ^69– 71^, by the local predator community ^72^, and by plants’ fungal and bacterial microbiomes ^73^. Taken together, the overall picture that emerges is a highly complex “phytochemical landscape” that dynamically shapes the co-evolution of plants and their herbivores ^74– 76^. Moving forward, this new appreciation of the integrative nature of trait-mediated plant-herbivore interactions suggests that plant functional traits are the key to understanding food-web interactions and ultimately ecosystem processes ^77^.

## Herbivore diversity and ecosystem function

The importance of species and functional diversity for maintaining healthy, resilient ecosystems is now widely recognized ^78– 80^. Although the field of biodiversity-ecosystem function is still dominated by plant-specific studies, consumer diversity is also clearly important for ecosystem function ^81– 83^. Herbivore diversity has strong impacts on many aspects of primary producer communities, such as primary production, plant diversity, and consumption of producer biomass ^84– 89^. Although it is not clear whether these patterns are due to complementarity among herbivore species or the idiosyncratic importance of individual species ^90^, the loss of herbivore diversity clearly impacts a variety of ecosystem functions.

More recent work has tried to integrate diet with other aspects of herbivore ecology, including movement, population growth, and predation risk. This has led to a more integrative understanding of herbivore complementarity and often finds higher levels of functional diversity among herbivore species than diet alone suggests. For example, herbivorous fishes on coral reefs often have similar diets but generally live in different habitats, feed from different substrates, and forage at different spatial scales ^91– 94^. These subtle differences in habitat selection and foraging range may influence how different herbivore species impact reef algal dynamics and competitive interactions between corals and algae, re-emphasizing that functional trait diversity is an important consideration in ecosystem-function studies ^95^.

Furthermore, both synthetic analyses and empirical work suggest that biodiversity supports multifunctionality in ecosystems ^79, 81^. In other words, in addition to influencing primary production, diversity influences ecosystem processes such as nutrient retention, nutrient cycling, and decomposition rates, and it is important to consider as many of these interconnected processes as possible ^96^. For example, Lefcheck *et al.* ^79^ used data from 94 biodiversity-ecosystem function experiments to test how species diversity impacts ecosystem multifunctionality across a range of taxa, trophic levels, and habitats. The authors showed that the effects of diversity on ecosystem multifunctionality (that is, the number of ecosystem processes surpassing a critical threshold of function) grew generally stronger the more functions that were considered, with consistent impacts across aquatic and terrestrial habitats. Perhaps most strikingly, the positive effects of plant diversity tapered off and even became negative at higher thresholds, whereas herbivore diversity had strong positive effects on multiple ecosystem processes even at the highest threshold levels. This result may stem from the fact that consumers often use a greater variety of resources and likely exhibit more complex behavior than plants ^97^ (but see 98). It also emphasizes conceptual predictions that consumer diversity might have stronger impacts on ecosystem function when compared with producer diversity ^99^.

### New technologies for understanding herbivore diversity: DNA metabarcoding

Determining herbivore diet breadth, and thus functional diversity, is challenging and difficult in many systems. Fortunately, this problem is becoming more tractable with recent technological advances. For example, dietary characterizations typically result from careful, time-consuming observations of herbivore feeding and behavior ^91, 100, 101^. However, visual observations are often limited by the ability to discriminate the actual species being consumed in mixed plant communities, the ability to observe nocturnal foraging, and difficulty in determining whether plant use is related to diet or habitat or both ^101^. To solve some of these problems, visual identification of plant fragments in either gut contents or dung is a common tool for a range of consumers ^102, 103^. However, identification of plants and other material is unequal across herbivores with different digestive physiologies, and smaller diet items often are resolvable only to functional group or family, and rare but potentially important diet items are typically missed entirely ^23^.

The development of DNA metabarcoding of herbivore gut contents may help quantitatively resolve herbivore diets, revealing previously cryptic aspects of functional diversity, complementarity, and niche partitioning ^104, 105^. DNA metabarcoding outperforms many traditional analyses in resolving diet identification ^23, 106^ and provides quantitative estimates of relative consumption of different foods and captures rare diet items ^23, 107, 108^. Furthermore, DNA metabarcoding has revealed previously cryptic functional differences among sympatric herbivore species using largely the same habitat. For example, Kartzinel *et al.* ^22^ used metabarcoding to examine diet niche partitioning for seven large mammalian herbivores—the African savanna elephant, impala, two species of zebra, buffalo, domestic cattle, and dik-dik—all co-occurring in a Kenyan savanna with over 100 plant species as potential diet items. Barcoding revealed that diets differed considerably across all levels of comparison. For example, grazers such as zebra consumed more than 99% grasses, mixed-feeding impala consumed about 35% grasses, and strict browsers such as dik-dik consumed less than 1% grass. Barcoding even revealed relatively fine-scale differences across ecologically similar grazer species (for example, the Grevy’s versus Plains zebra and African buffalo versus domestic cattle). Remarkably, although the two species of zebra consumed nearly identical proportions of grasses, they relied on very different suites of grass species, a finding that would have been difficult to detect using traditional methods of diet analysis.

Barcoding technology could be especially important for unpacking the ecology of previously intractable species, including large generalist herbivores with broad home ranges across a variety of habitats (for example, terrestrial ungulates), for herbivores that feed on visually cryptic species (for example, algal-feeding fishes), for morphologically similar herbivores (for example, rolled-leaf beetles ^109, 110^), and for revealing spatiotemporal patterns in diet. Given the continually shrinking cost of high-throughput, next-generation sequencing, these types of large-scale, long-term projects will hopefully become increasingly common.

## Too scared to eat? context-dependent effects of predation risk on plant-herbivore interactions

Risk of predation can alter herbivore foraging behavior and subsequently impact the abundance, distribution, or diversity of primary producers. This cascade is evident across a variety of ecosystems, including rocky intertidal habitats ^111^, seagrass beds ^112^, coral reefs ^113, 114^, freshwater ponds and streams ^115, 116^, old fields ^117, 118^, temperate grasslands and forests ^72, 119– 121^, and African savannas ^122^. Ultimately, predation risk may even drive patterns of carbon sequestration in heavily vegetated habitats ^123, 124^, as the mere risk of predation alters levels of plant consumption and the standing stock of carbon trapped in plant biomass.

**Figure 2.**
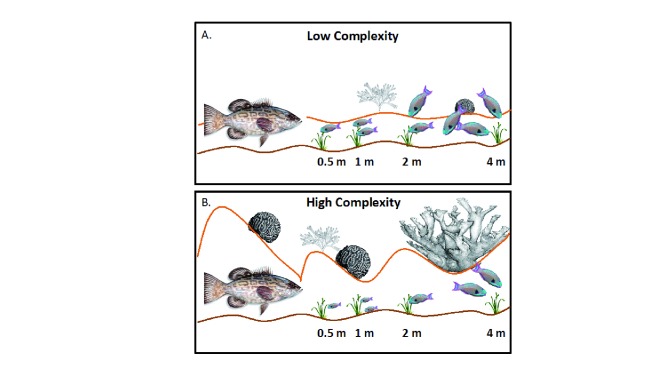
The impact of risk of predation on plant-herbivore interactions often depends on many context-dependent factors such as habitat complexity. Catano *et al.* ^127^ used taxidermy decoy black grouper to manipulate risk in areas of low and high complexity on a Caribbean coral reef. They measured rates of herbivory and quantified bites of common parrotfishes and surgeonfishes at increasing distances from the decoy grouper. In areas of low complexity ( **A**), overall rates of herbivory by parrotfishes and surgeonfishes are lower than in control areas without grouper decoys (not shown) but are significantly higher than in areas of higher complexity ( **B**). In areas of higher complexity, rates of herbivory are significantly lower at closer distances to the decoy grouper than in the low-complexity areas. However, feeding by the smallest herbivorous fishes was greatest at closer distances in the higher-complexity areas, potentially due to the smaller fishes being less vulnerable to large grouper and also due to having more refuge from predation in the complex habitats. Illustration courtesy of Laura Catano.

More recent work has focused on how context-dependent factors such as habitat complexity, predator identity, herbivore identity, body size, and prey hunger level can influence the cascading effects of risk aversion ^125, 126^. For example, a study of herbivorous fishes on a coral reef showed that decoy predatory fishes suppressed herbivory by parrotfishes and surgeonfishes significantly more in high-complexity areas and at distances farther from the decoy when compared with low-complexity areas ( Figure 2), likely due to decreased visibility and perceived escape ability in more complex areas ^127^. Furthermore, smaller herbivorous fishes were more willing to forage closer to the decoys than were larger fishes, especially in more complex areas, possibly because they were less of a target for the much larger predators and because complex areas provided more small refuges for smaller individuals. Thus, there may be strong interactions in habitat complexity and body size in shaping patterns of risk-driven herbivory.

Species identity is likely also an important context-dependent driver of how predation risk impacts patterns of herbivory across the landscape. For example, in the species-rich guild of ungulate herbivores in African savannas, different species such as giraffe, zebra, and impala exhibit different responses to increased density of woody vegetation, typical hiding spots for predators like lions and leopards ^128, 129^. This differential habitat selection due to risk aversion interacts with forage quality and quantity, resulting in heterogeneous impacts of herbivores across the landscape. Wildebeest and impala, for example, exert strong top-down control on plant communities in different parts of the savanna on the basis of how predation risk and food resources shape their habitat selection ^130^. Interestingly, increasing climate variability may alter the fear landscape. In normal rainfall years, herbivores such as zebra and gazelles favored areas with fewer trees and higher visibility to detect predators. But, during a drought, these same herbivores frequented areas with more grass regardless of tree density and predation risk ^131^. Thus, these herbivores were trading safety for food during stressful times, showing that variations in climate and resource levels strongly influence the landscape of fear.

The role of predator foraging behavior (for example, sit-and-wait versus roving) has also emerged as an important topic for understanding the impact on herbivore behavior and ultimately their impact on plant communities. Schmitz ^118^ used a three-year experiment in grassland mesocosms to show that actively hunting spiders reduced grasshopper abundance, resulting in increased plant species diversity and enhanced aboveground net primary production and nitrogen mineralization rate. In contrast, sit-and-wait ambush spiders mostly impacted grasshopper behavior, not density, which had the opposite effect on plant communities and ecosystem processes. Sit-and-wait predators often result in larger shifts in herbivore behavior than do more mobile, yet more unpredictable, predators, but it is unclear how strongly and how often this effect filters down to influence plant communities ^132– 134^.

Advances in technology, specifically in telemetry, satellite imagery, and remote sensing, have opened up myriad ways to better document and understand the cascading effects of predation risk ^24, 135^. For example, in an African savanna, Ford *et al.* ^122^ used GPS telemetry, satellite imagery, and elegant small-scale experiments to show that the risk of predation from leopard and wild dog drove impala into areas with lower woody cover and thus a lower probability of encountering predators. In turn, this led to a suppression of the palatable tree *Acacia brevispica* in areas of high impala abundance while facilitating the abundance of *Acacia etbacia*, a well-defended, thorny species. Interestingly, the effects of predation risk can even be seen from space. For example, in coral reef ecosystems, the halos of bare space that herbivorous fishes create around patch reefs, in large part due to predation risk in the open water, are clearly evident even in relatively low-resolution Google Earth images ^136^. These halos tend to be smaller in areas of high predation risk but lower, or even nonexistent, in areas of low predation risk. Further development and cost reductions of these technologies will help reveal aspects of risk-driven trophic cascades that have been hidden to date.

## Plant-herbivore interactions in an era of climate change

Recent evidence suggests that climate change is happening 10 times faster than at any time in the last 65 million years ^137^, having profound consequences for life on earth. The pace of climate change varies considerably across ecosystems ^138^, but one of the most striking aspects of numerous recent studies is the relatively rapid ecological and evolutionary responses of plant-herbivore interactions to our warming climate. Hundreds of species of crop pests and pathogens, for example, have moved poleward at an average of 2.7 km/year since 1960 in the Northern Hemisphere ^139^, essentially matching the observed temperature increases ^140^. In many cases, herbivores appear to be responding faster to climate change than their host plants ^141, 142^, leading to altered selective pressures and novel ecological interactions in their new ranges. The habitat specialist mangrove crab ( *Aratus pisonii*), for example, is moving northward on the eastern Atlantic coastline by 6.2 km/year ^143^, far outpacing estimated mangrove migration rates of 1.3 to 4.5 km/year ^144^. In mangrove habitats, *A. pisonii* is an important herbivore and closely tied to mangrove trees ^145^, but the lack of their hosts in salt marshes leads to altered behavior and habitat selection, diet, size, and reproductive traits ^146, 147^.

Similarly, climate change has strengthened the flow of ocean currents, leading to “oceanic hotspots” and the expansion of the ranges of many tropical fish species into more temperate regions. The “tropicalization” of these temperate ecosystems has already resulted in overgrazing on temperate macroalgal communities in the Mediterranean, Japan, the Gulf of Mexico, Australia, and South Africa ^148^. Indeed, the sudden arrival of tropical herbivorous fishes has essentially eliminated kelp recruitment on some temperate Australian reefs, leading to potentially persistent phase-shifts away from fleshy-kelp communities toward algal turf-dominated reefs ^149^. A similar process of “phenological mismatch” has happened in the temperate boreal zone, where warmer winters have reduced snowpack, leading to increased herbivory on aspen and other woody species ^150^. Interestingly, warmer winters have the opposite effect in the High Arctic, where increasing amounts of rain during extremely warm winters have hardened the snowpack and reduced availability of winter forage availability for overwintering vertebrate herbivores. As a consequence, these extreme events can cause widespread herbivore population crashes that ripple through to predator populations ^151, 152^.

In most of these cases, changes in herbivore behavior allow for rapid responses to climate change (for example, ^153^), but there is some question over whether there is subsequent genetic changes that could allow for adaption to climate change. One example of rapid evolution to climate change is that of the winter moth ( *Operophtera brumata*), in which egg hatching date has closely tracked phenological changes in budburst of its host, the oak *Quercus robur* ^154^. However, herbivores with longer generation times may be less able to respond adaptively to a rapidly changing climate. For example, roe deer in Western Europe are experiencing earlier springs, but because they have relatively inflexible mean birthing dates, fawns are increasingly born when high-quality early-spring vegetation is becoming less abundant ^155^, having potential long-term consequences on their abundance and distribution.

**Figure 3.**
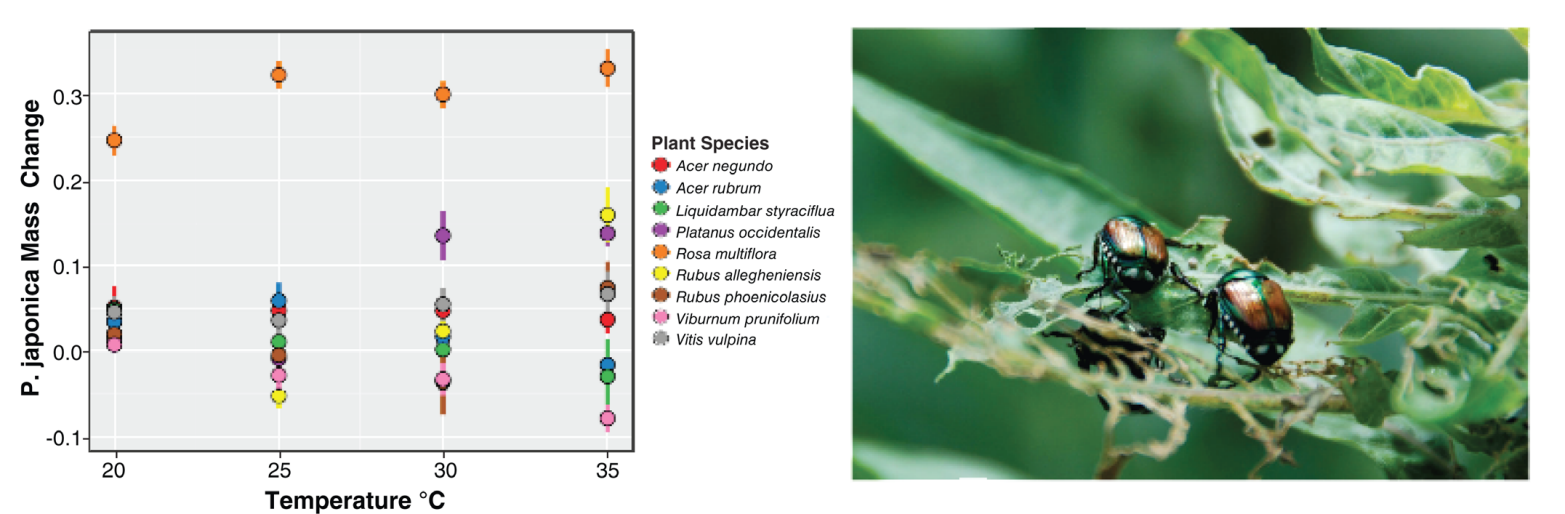
Climate warming may have idiosyncratic effects on plant-herbivore interactions, depending on the species involved Lemoine *et al.* ^162^ showed that increasing temperature affects growth rates of Japanese beetles ( *Popilla japonica*, at right skeletonizing an *Oenothera biennis* leaf) differently depending on the plant species they are feeding on. For example, Japanese beetle growth increased with temperature on plants such as *Platanus occidentalis* (purple points) but declined on species such as *Viburnum prunifolium* (pink points). These data highlight the difficulty in predicting the impact of climate warming on plant-herbivore interactions. Data redrawn after Lemoine *et al.* ^162^. Image from Dejeanne Doublet.

The main drivers of climate change—increasing carbon dioxide (CO _2_) and temperature—also fundamentally alter the physiology and metabolism of both herbivores and plants ^156^. Metabolic theory predicts that warmer temperatures should lead to elevated metabolism in ectothermic consumers resulting in increased feeding rates ^157, 158^. Yet experimental work shows that feeding rates of insect herbivores can increase, decrease, or remain unchanged at higher temperatures ^159– 162^ and that responses vary even for a single herbivore species among different host-plant species ^161, 162^ ( Figure 3). In other cases, there is a striking interaction with elevated temperatures and the intake of plant secondary compounds, and there is some evidence for enhanced toxicity of compounds at higher temperatures ^163^, but again with considerable variability among different species ^162^.

Climate change, particularly increasing CO _2_ levels, may also impact how plants allocate resources to growth, defense, and reproduction, which may profoundly influence herbivore feeding behavior. Elevated CO _2_ may lower plant nutritional quality, particularly nitrogen density, slowing herbivore growth and reproduction and forcing herbivores to increase consumption rates or shift diets ^164– 166^. However, the responses of insect herbivores to elevated CO _2_ vary widely, and some taxa like Lepidoptera decline in performance, on average, with increased CO _2_ while Homopteran performance increases ^166^. Increasing CO _2_ also alters the production of chemical defenses in plants, although the changes in defensive chemistry are often idiosyncratic depending on the plant taxa and chemical compounds ^167^. Flavonoids, phenolics, and condensed tannins increase, on average, with elevated CO _2_ while terpene production declines ^166^. For example, elevated CO _2_ caused reduction in the production of cardenolide defensive chemicals in the milkweed ( *Asclepias syriaca*), yet CO _2_ stimulated production in physical defenses (for example, leaf toughness and latex production), which could have balanced the effect of reduced chemical defenses on herbivores ^168^. In the future, work examining interactions between temperature and increased CO _2_ on plant-herbivore interactions at meaningful ecological scales is sorely needed, as is work that examines the evolutionary consequences of climate change.

## Conclusions: conservation of plant-herbivore interactions in the Anthropocene

Species are being lost from many ecosystems at an alarming rate, and large vertebrates often are the first to go ^169, 170^. Although predator loss is often emphasized, herbivores are also being continually lost to extinction ^171, 172^, having cascading impacts on the integrity of entire ecosystems ^173– 176^. Sadly, parallels can be drawn between the contemporaneous loss of herbivores with the vast changes that occurred following the Pleistocene megafaunal extinction, when many continents lost most of their large consumers, resulting in “no-analog” plant communities with novel suites of interactions and presumably altered ecosystem functionality ^177– 179^.

In contrast, the opposite effect is also a prevailing problem in many ecosystems. For example, explosive population growth of white-tailed deer ( *Odocoileus virginianus*) resulting from loss of predators and human-altered habitat has led to widespread overbrowsing and loss of plant diversity in temperate forests in North America ^179– 183^, a situation that may take decades to reverse ^184^. White-tailed deer are particularly emblematic of the difficulties inherent in managing plant-herbivore interactions within the context of ecosystem conservation. For example, numerous studies show that the negative effects of deer can be ameliorated and even reversed at densities approaching their historical levels (for example, ^180, 185, 186^), but there remains significant opposition to implementing meaningful hunting and culling programs aimed at reducing deer densities ^187^. These intransigent problems demonstrate that in many cases we do not lack the scientific information to exert meaningful differences, only the collective willpower.

Finally, we note that the study of plant-herbivore interactions continues to be a leading light in ecology and evolution, demonstrating the power of applying new technologies and multiple perspectives to resolving long-standing uncertainties. These newfound approaches show that in many cases the world is even more complex than we once thought ^22, 109^. Thus, our challenge is to find conservation solutions that accurately reflect the pervasive impacts of plant-herbivore interactions across broad temporal and spatial scales, preserving ecosystem multifunctionality and sustainability for future generations.
